# Cytogenetics of insects in the era of chromosome-level genome assemblies

**DOI:** 10.18699/vjgb-25-26

**Published:** 2025-04

**Authors:** V.A. Lukhtanov, E.A. Pazhenkova

**Affiliations:** Zoological Institute of the Russian Academy of Sciences, St. Petersburg, Russia; University of Ljubljana, Ljubljana, Slovenia

**Keywords:** chromosome, karyotype, chromosomal rearrangements, telomere, meiotic drive, recombination, sex chromosomes, inversion, synteny, хромосома, кариотип, хромосомные перестройки, теломера, мейотический драйв, рекомбинация, половые хромосомы, инверсии, синтении

## Abstract

Over the past few years, a revolution has occurred in cytogenetics, driven by the emergence and spread of methods for obtaining high-quality chromosome-level genome assemblies. In fact, this has led to a new tool for studying chromosomes and chromosomal rearrangements, and this tool is thousands of times more powerful than light microscopy. This tool has revolutionized the cytogenetics of many groups of insects for which previously karyotype information, if available at all, was limited to the chromosome number. Even more impressive are the achievements of the genomic approach for studying the general patterns of chromosome organization and evolution in insects. Thus, it has been shown that rapid transformations of chromosomal numbers, which are often found in the order Lepidoptera, are most often carried out in the most parsimonious way, as a result of simple fusions and fissions of chromosomes. It has been established that these fusions and fissions are not random and occur independently in different phylogenetic lineages due to the reuse of the same ancestral chromosomal breakpoints. It has been shown that the tendency for chromosome fissions is correlated with the presence in chromosomes of the so-called interstitial telomeres, i. e. telomere-like structures located not at the ends of chromosomes, but inside them. It has been revealed that, in most insects, telomeric DNA is not just a set of short repeats, but a very long sequence consisting of (TTAGG)n (or other telomeric motifs), regularly and specifically interrupted by retrotransposons, and the telomeric motifs are diverse in terms of their length and nucleotide composition. The number of high-quality chromosome-level genome assemblies available for insects in the GenBank database is growing exponentially and now exceeds a thousand species. Therefore, the exceptional prospects for using genomic data for karyotype analysis are beyond doubt.

## Abstract (ru)

За последние несколько лет в цитогенетике произошли серьезные изменения, связанные с разработкой и распространением методов получения высококачественных хромосомных сборок полных геномов. Фактически это привело к появлению нового инструмента для изучения хромосом и хромосомных перестроек, мощность которого многократно превосходит возможности световой микроскопии. Использование этого инструмента революционизировало частную цитогенетику многих групп насекомых, для которых ранее информация о кариотипах, если она была вообще, ограничивалась элементарным подсчетом числа митотических или мейотических хромосом. Цель данного краткого обзора – обобщение достижений сравнительной и эволюционной цитогенетики насекомых, которые были получены на основании биоинформатического анализа хромосомных сборок полных геномов. С помощью этого подхода было показано, что в процессе быстрой хромосомной эволюции у чешуекрылых (отряд Lepidoptera) преобразования хромосомных чисел чаще всего осуществляются наиболее парсимониальным способом: в результате простых слияний и разделений хромосом. Установлено, что эти слияния и разделения не случайны и могут осуществляться в разных филогенетических линиях за счет повторного использования одних и тех же предковых хромосомных точек разрыва. Тенденция к разделениям хромосом скоррелирована с наличием в хромосомах так называемых интерстициальных теломер – теломероподобных структур, расположенных не на концах хромосом, а внутри них. При изучении теломерных регионов выявлено, что у большинства насекомых теломерная ДНК – это не просто набор коротких повторов, а очень длинная последовательность, состоящая из (TTAGG)n (или других мотивов), регулярно и специфически прерываемая ретротранспозонами, а сами теломерные мотивы чрезвычайно разнообразны по длине и нуклеотидному составу. Число высококачественных хромосомных сборок геномов насекомых, доступных в базе данных GenBank, растет в геометрической прогрессии и уже превышает тысячу видов. Поэтому исключительные перспективы использования хромосомных сборок геномов для анализа кариотипов не вызывают сомнений.

## Introduction

Progress in science is often driven by new research methods. In the field of genetics, one such fundamentally new approach, which has given a powerful impetus to the development of the entire discipline, is the DNA sequencing procedure (Heather, Chain, 2016). High-throughput sequencing methods, along with advances in bioinformatics and the development of the Hi-C DNA analysis protocol (Lieberman-Aiden et al., 2009), have recently led to a breakthrough technology for obtaining genome assemblies at the chromosome level (Dudchenko et al., 2017). This methodology has revolutionized comparative cytogenetics, stimulating the emergence of a large number of works devoted to the structure of chromosomes, patterns of chromosomal changes in evolution and role of these changes in speciation. In fact, this new technique has switched the attention of many biologists, especially bioinformaticians, from the analysis of nucleotide substitutions to the analysis of structural changes in DNA, enhancing and complementing the work previously done with a microscope.

The aim of this brief review is to analyze and summarize the advances made in comparative and evolutionary cytogenetics of insects using bioinformatic analysis of chromosome level genome assemblies.

## The main stages of insect cytogenetics

Although the karyotypes of some model insect species, such as the midge Chironomus plumosus and the silkworm Bombyx mori, have been studied in great detail (Kiknadze et al., 1991, 2016; Yoshido et al., 2005), for non-model species, information on karyotypes, if available at all, is often limited to the estimation of the diploid (or haploid) number of chromosomes, an approximate description of the size characteristics of individual chromosomes and, less often, individual chromosome arms in the form of a centromeric index (Peruzzi, Eroğlu, 2013). It should be noted that obtaining the latter characteristic is, in principle, impossible for representatives of many insect orders, for example, for butterflies (Lepidoptera) and bugs (Hemiptera), since they have holocentric chromosomes, that is, they do not have a localized centromere (Mandrioli, Manicardi, 2020). Such a low average level of insect cytogenetics is largely due to the objective difficulties of studying chromosomes using a microscope: the sizes of chromosomes are often at the limit of the resolving power of light microscopy

It is therefore not surprising that in the history of cytogenetics, beginning with its inception in the 19th century, attempts have been made to increase the resolving power of cytogenetic analysis. The first stage in the history of cytogenetics can be called the era of chromosome numbers. It arose in the second half of the 19th century, when the first descriptions and images of karyotypes containing the correct determination of the number of chromosomes appeared (e. g., Henking, 1890). The heyday of this era came in the first half of the 20th century, when the study of karyotypes became a mass phenomenon (Beliajeff, 1930; White, 1973).

Significant progress in cytogenetic research was associated with the emergence and widespread use in the second half of the 20th century of methods of differential staining of chromosomes, such as C-banding (Pardue, Gall, 1970) and G-banding (Seabright, 1971). Cytogenetics entered the era of chromosome banding. Almost simultaneously, even more powerful methods of cytogenetic analysis appeared and were developed in parallel, based on the use of the FISH method (Gall, Pardue, 1969; Langer-Safer et al., 1982) and its modifications, such as BAC-FISH (BAC Resource Consortium 2001; Yoshido et al., 2005) and chromosome painting (Schrock et al., 1996; Speicher et al., 1996). This has led to stunning advances in cytogenetics of many groups of organisms, especially vertebrates (Ferguson-Smith, Trifonov, 2007; Graphodatsky et al., 2011). As for insects, with the exception of some model species (Yoshido et al., 2005), this progress has affected them to a lesser extent. Of course, light microscopes have become much better, and the resulting images of karyotypes have become much clearer compared to what they were 100 years ago. In addition, the GISH method has made it possible to effectively detect sex chromosomes (Fukova et al., 2005; Šíchová et al., 2015). Despite this, the cytogenetics of many insect groups, for example, most families of Lepidoptera, is still at the stage of elementary counting of chromosome numbers (Pazhenkova, Lukhtanov, 2023a).

## Chromosome level genome assemblies: a new tool for studying karyotypes

A revolution in the field of karyotype studies has occurred over the past six-eight years. Modern approaches to genome analysis based on obtaining long reads and using Hi-C technology (Dudchenko et al., 2017) make it possible to obtain chromosome level genome assemblies, in which all or at least most of the chromosomes are read from telomere to telomere (Miga et al., 2020; The Darwin Tree…, 2022; Zhang et al., 2023).

Currently, high-quality chromosome level genome assemblies have been obtained for representatives of most insect orders: fleas (Siphonaptera) (Driscoll et al., 2020), stoneflies (Plecoptera) (Dixon et al., 2023), dipterans (Diptera) (Zamyatin et al., 2021; Reinhardt et al., 2023), beetles (Coleoptera) (Van Dam et al., 2021; Huang et al., 2022), springtails (Collembola) (Jin et al., 2023), stick insects (Phasmatodea) (Lavanchy et al., 2024), Hymenoptera (Sun et al., 2021), mayflies (Ephemeroptera) (Farr et al., 2023), Hemiptera (Biello et al., 2021; Mathers et al., 2021; Chen H. et al., 2022; Wang et al., 2024), Orthoptera (Li R. et al., 2024), Trichoptera (Ge et al., 2024), Psocoptera (Feng et al., 2022), Neuroptera (Wang et al., 2022), Odonata (Patterson et al., 2024), Thysanoptera (Yingning et al., 2024), Dermaptera (https://www.ncbi.nlm. nih.gov/datasets/genome/GCA_963082975.1/) and a large number of Lepidoptera species (Mackintosh et al., 2022a; Gauthier et al., 2023; Wright et al., 2024). These assemblies contain information on the haploid number of chromosomes and the size of each chromosome, measured in the number of base pairs. Almost always, there is also information on the presence and size of the sex chromosome X (Z for Lepidoptera and caddisflies, in which females are the heterogametic sex) and, less often, the sex chromosome Y (W for Lepidoptera).

**Fig. 1.**
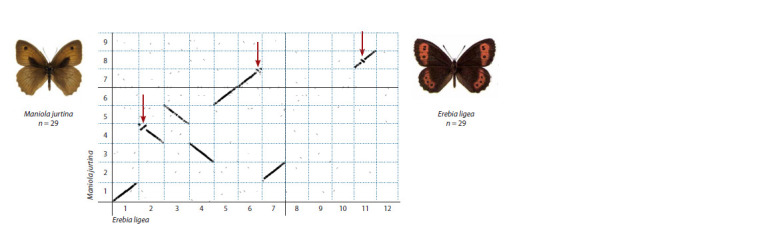
Comparison of the genomes of the butterflies Maniola jurtina and Erebia ligea based on chromosome assemblies. The butterflies M. jurtina and E. ligea have 29 chromosomes in the haploid set. The first 12 chromosomes of E. ligea are mapped on the abscissa axis. The first nine chromosomes of M. jurtina are mapped on the ordinate axis. The diagonals on the graph show the regions of macrosynteny. Inversions are marked with red arrows. It is seen that chromosome 1 of M. jurtina is homologous to chromosome 1 of E. ligea, chromosome 2 of M. jurtina is homologous to chromosome 7 of E. ligea, etc. (according to: Pazhenkova, Lukhtanov, 2023b).

The use of chromosome level genome assemblies has actually led to the emergence of a new methodology and a new tool for studying chromosomes and chromosomal rearrangements. The resolution power of this tool significantly exceeds the capabilities of light microscopy. The basis of the methodology is to obtain pairwise or multiple alignments of chromosome assemblies of different species. These alignments are usually presented in the form of circular plots (Krzywinski et al., 2009). Another analysis option is to obtain pairwise comparisons presented in the form of dot plots (Li H., 2018), in which the nucleotide sequences of individual chromosomes are plotted along the abscissa and ordinate axes, starting with the first, largest chromosome (Fig. 1). Such graphs clearly demonstrate macrosyntenic regions and identify chromosome fusions/fissions, as well as chromosomal inversions. The latter are visible on the graph as segments that are perpendicular to the main diagonals (Fig. 1).

The use of this tool has revolutionized cytogenetics of many insect groups, for which karyotype information, if any, was previously limited to elementary counts of mitotic or meiotic chromosomes. However, even more impressive are the achievements of the genomic approach to the study of general patterns of chromosome organization and karyotype evolution, including the analysis of chromosomal rearrangements and chromosomal syntenies (Biello et al., 2021; Mathers et al., 2021; Sun et al., 2021; Van Dam et al., 2021; Höök et al., 2023; Hundsdoerfer et al., 2023; Wright et al., 2024), as well as the reconstruction of ancestral karyotypes (Chen X. et al., 2023; Wright et al., 2024). Chromosome level genome assemblies have been used to study meiotic drive (Reinhardt et al., 2023; Boman et al., 2024), sex chromosome evolution (Mackintosh et al., 2022b; Berner et al., 2023; Höök et al., 2023, 2024), interspecific transfer of chromosomal inversion during interspecific hybridization (Seixas et al., 2021), the role of chromosomal rearrangements in the evolution of recombination frequency (Näsvall et al., 2023), and to identify genomic coordinates for breakpoints that give rise to chromosomal rearrangements (Zamyatin et al., 2021).

## Chromosomal conservatism and rapid karyotypic evolution

In our work (Pazhenkova, Lukhtanov, 2023a), we used the analysis of chromosome level genome assemblies to solve one of the mysteries of evolutionary cytogenetics. It is known that the chromosome numbers of many insects are conservative and remain unchanged or with minimal changes for tens and hundreds of millions of years (White, 1973). For example, in the order Lepidoptera (butterflies and moths), the ancestral haploid chromosome number n = 31 has been preserved for 200 million years, although n = 30 is often found in some species along with n = 31. In the blue butterflies (the family Lycaenidae), the haploid number n = 24 predominates, although n = 23 is often found (Robinson, 1971). This suggests that large chromosomal rearrangements are rare in the evolution of the order Lepidoptera. At the same time, in some genera of butterflies, there are explosions of karyotypic variability,and chromosome numbers change dramatically in a very short time, for example, during the divergence of two closely related species (White, 1973). The chromosomal mechanisms of such rapid karyotypic evolution were unclear. In addition, it was unclear how real the phenomenon of chromosomal conservatism itself was, since the preservation of the ancestral chromosome number does not exclude intra-chromosomal rearrangements 

Analysis of chromosome level genome assemblies in multiple Lepidoptera species showed that in the evolutionary phase of chromosomal conservatism, most autosomes are indeed stable. However, this does not apply to the sex chromosome Z. Fusions of the Z chromosome with one of the autosomes, independently occurring in different evolutionary lineages, lead to multiple variants of the NeoZ chromosome and a decrease in the haploid number by one unit.

**Fig. 2.**
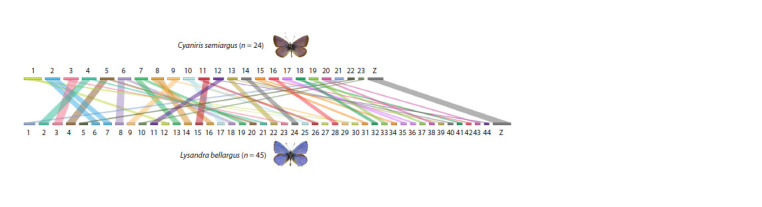
Schematic representation of chromosomes and regions of macrosynteny in the karyotypes of the butterflies Cyaniris semiargus (n = 23+Z) and Lysandra bellargus (n = 44+Z). The karyotype of L. bellargus differs from the karyotype of C. semiargus by chromosome fissions that have occurred in 21 out of 23 autosomes (according to: Pazhenkova, Lukhtanov, 2023a).

**Fig. 3.**
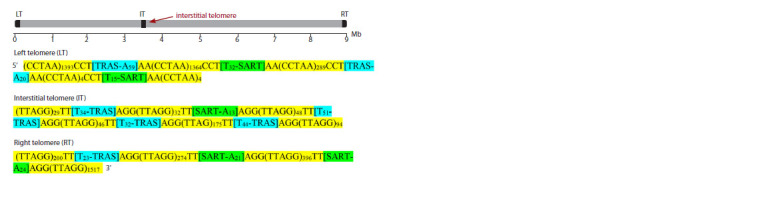
Telomere and interstitial telomere structure in chromosome 38 of Lysandra bellargus Each telomere is a long array (CCTAA)n/(TTAGG)n (yellow) interspersed with retrotransposable TRAS (blue) and SART (green) elements. The TRAS and SART elements have long An/Tn and Tn/An tails and are specifically inserted between CCT/ AGG and AA/TT nucleotides of the TTAGG motif (according to: Pazhenkova, Lukhtanov, 2023a).

As for the explosive karyotypic evolution, the most rapid changes in chromosome numbers are carried out in a parsimonious way: as a result of simple fusions and fissions of chromosomes (Fig. 2). Moreover, these fusions and fissions are not random and can be carried out in different phylogenetic lineages due to the repeated use of the same ancestral chromosomal breakpoints (Pazhenkova, Lukhtanov, 2023a). It should also be noted that the tendency for breaks is correlated with the presence of the so-called interstitial telomeres in chromosomes, i. e. telomere-like structures located not at the ends of chromosomes, but inside them (Fig. 3).

## Telomeric DNA of insects

It is believed that in most insects, telomeric DNA consists of a canonical five-letter TTAGG motif, which is repeated hundreds and thousands of times at the ends of chromosomes (Kuznetsova et al., 2020). However, the analysis of telomeric DNA in 220 insect species in our studies (Lukhtanov, 2022; Lukhtanov, Pazhenkova, 2023), as well as other works that appeared in parallel (Zhou et al., 2022; Fajkus et al., 2023), showed that in addition to the canonical TTAGG motif, insects contain a large number of other variants of telomeric repeats, the length of which varies from 1 to 11 nucleotides (see the Table).

**Table 1.**
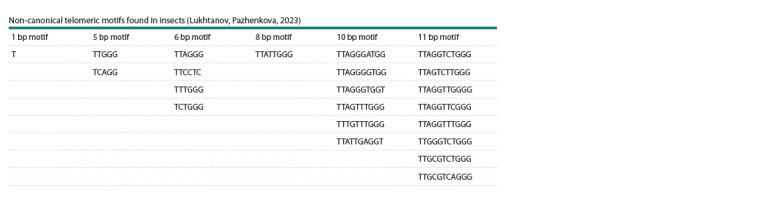
Non-canonical telomeric motifs found in insects (Lukhtanov, Pazhenkova, 2023)

**Fig. 4.**
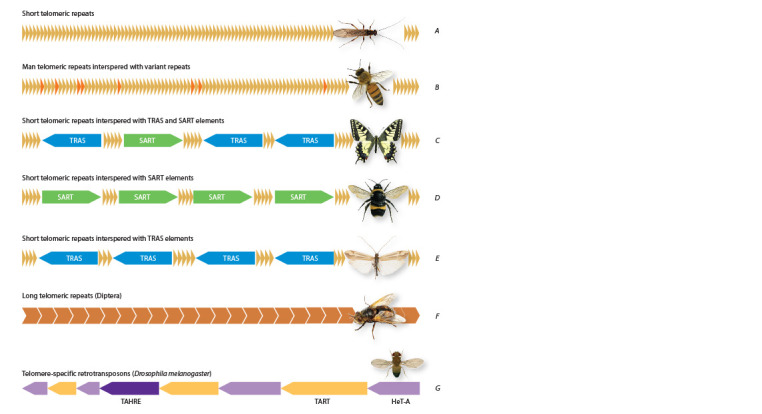
Types of telomeric DNA organization in insects. A – short repeats; B – main short repeats interspersed with variant short repeats, known in the honey bee Apis mellifera (Wallberg et al., 2019); C – main short repeats with insertions of telomere-specific non-LTR retrotransposons of the TRAS and SART families (found in Lepidoptera); D – main short repeats (5–11 bp) with insertions of telomere-specific non-LTR retrotransposons of the SART family (found in most studied species of Hemiptera, Coleoptera and many Hymenoptera); E – main short repeats with insertions of telomere-specific non-LTR retrotransposons of the TRAS family (found in Trichoptera); F – long (173–381 bp) repeats (found in Diptera); G – telomere-specific non-LTR retrotransposons of the HeT-A, TAHRE and TART families (found in Drosophila melanogaster) (Biessmann et al., 2000; Casacuberta, Pardue, 2003).

Even more intriguing is the fact that the vast majority of insects have telomeres with a complex multilayer structure (Lukhtanov, Pazhenkova, 2023). In these telomeres, blocks of short telomeric motifs are regularly interrupted by retrotrans posons that are specifically embedded in repeats of both the canonical TTAGG motif (Fig. 3) and other non-canonical motifs. In our opinion, such a structure indirectly indicates the presence of two parallel mechanisms for maintaining telomere length during cell divisions in insects: the classical telomerase mechanism and a mechanism based on transpositions. In general, insects are characterized by a great diversity in the organization of telomeric DNA, which is summarized in Figure 4.

## Prospects for the use of chromosome level genome assemblies in cytogenetics

The number of high-quality chromosome level genome assemblies available in the GenBank database is growing exponentially due to the activities of various laboratories, and primarily The Wellcome Sanger Institute in the UK (The Darwin Tree…, 2022). Currently, GenBank contains information on chromosome assemblies of genomes (including determination of the haploid number of chromosomes) for 1,118 insect species (https://www.ncbi.nlm.nih.gov/datasets/ genome/?taxon=50557, dated May 15, 2024). Thus, it can already be stated that over the past three-four years, the number of new insect karyotypes, obtained using bioinformatic analysis of genomes, is comparable to or even exceeds the number of karyotypes studied using routine cytogenetic analysis. In addition, chromosomal assemblies of genomes carry several orders of magnitude more information about the obtained karyotypes.

The successes of the genomic approach discussed above do not mean that classical cytogenetics, which provides information on the real spatial configurations of chromosomes, should be discounted. Classical cytogenetics is also needed to validate chromosome level genome assemblies, in particular to confirm the number of chromosomes (Pazhenkova, Lukhtanov, 2023a) and the structure of telomeric DNA motifs (Dalla Benetta et al., 2020; Stoianova et al., 2024). Examples of such validation reinforce the conclusion that chromosome level genome assemblies are a reliable source of information on karyotypes. For instance, for butterflies and moths (order Lepidoptera), information on chromosome assemblies, including determination of the haploid number of chromosomes, is available for 452 species (https://www.ncbi. nlm.nih.gov/datasets/genome/?taxon=7088, from May 15, 2024). For more than half of them, there are data on chromosome numbers obtained using light microscopy methods. Having compared these data, we found complete agreement in the haploid chromosome number calculations made using the bioinformatics approach and light microscopy methods (Pazhenkova, Lukhtanov, 2023a).

Thus, the exceptional prospects of using chromosome level genome assemblies for karyotype analysis are beyond doubt.

## Conflict of interest

The authors declare no conflict of interest.
